# Vesicovaginal Fistula and Hydronephrosis Post-radiation for Cervical Cancer: Challenges and Strategies in Management

**DOI:** 10.7759/cureus.63195

**Published:** 2024-06-26

**Authors:** Maged Almogahed, Jinguo Wang, Dawei Wu, Xin Gao, Salem Baldi

**Affiliations:** 1 Department of Urology, The First Bethune Hospital of Jilin University, Changchun, CHN; 2 Department of Clinical Laboratory Diagnostics, School of Medical Technology, Shaoyang University, Shaoyang, CHN

**Keywords:** postoperative care, cervical cancer, radiation therapy, hydronephrosis, vesicovaginal fistula (vvf)

## Abstract

This report meticulously examines a complex case involving a vesicovaginal fistula (VVF) and hydronephrosis following radiation treatment for stage IV cervical cancer. It highlights the challenges and nuanced treatment strategies essential for addressing such complications, particularly given the patient's comprehensive medical background, including her cancer treatment and subsequent radiation therapy. Through an in-depth case study, the report details the diagnostic process, emphasizing the role of advanced imaging techniques and clinical evaluations in detecting a VVF along with hydronephrosis - illustrating the delayed adverse effects of radiation therapy. The treatment strategy employed a laparoscopic surgical technique, coupled with a carefully devised postoperative care plan focusing on hydration and anti-inflammatory measures to counteract radiation cystitis and prevent further issues. This narrative not only showcases the complex challenges of managing complications such as VVF and hydronephrosis post-radiation but also underlines the critical need for personalized, interdisciplinary care approaches. These approaches strive to effectively treat cancer while prioritizing the patient's quality of life. Furthermore, the report adds valuable insights to the ongoing dialogue on improving care for individuals facing the enduring impacts of cancer treatments. It argues for a comprehensive approach that includes preventive measures, prompt diagnostic procedures, and customized care strategies to improve the health and quality of life for cancer survivors.

## Introduction

Vesicovaginal fistulas (VVF) represent a critical and socially impactful challenge in contemporary medicine, primarily due to the profound decline in patients' quality of life and subsequent social disadaptation. Traumatic events, including childbirth and obstetric or gynecological surgeries, account for 97% of vaginal fistula cases, underscoring the importance of addressing this issue in medical practice and research [1]. VVF develop in 85% of patients following gynecological surgeries, occur in 11% of cases post-childbirth, and result from radiation exposure in 4% of cases [1]. Additional risk factors encompass adjacent malignancies and multiple pelvic surgeries. Typically, patients manifest with incessant urinary leakage from the vagina. Should radiation be implicated, symptom onset might be postponed for years. Diagnosis primarily relies on clinical assessment, including pertinent history and physical examination, and is corroborated by bladder instillation of methylene blue, with further delineation through targeted imaging studies [2]. Surgical intervention is the cornerstone of treatment, offering both vaginal and abdominal approaches [3]. The vaginal route is favored for reducing blood loss, shortening hospital stays, and alleviating pain [3]. In this case, a 44-year-old woman with VVF, a history of stage four cervical cancer, and a decade of radiotherapy underwent surgical treatment for her fistula.

## Case presentation

A 44-year-old female, with a history of stage IV cervical cancer treated with radiation therapy 14 years ago, exhibited persistent urinary incontinence for over two months, which intensified a week prior to her admission on March 11, 2024. She was diagnosed with a vesicovaginal fistula, as confirmed by pelvic MRI and cystoscopy. The fistula, measuring 2 cm, was located near the bladder's base, surrounded by extensive scar tissue that rendered the bilateral ureteral openings invisible. This diagnosis underscores a complex clinical scenario that necessitates thorough analysis and individualized treatment planning.

**Figure 1:**
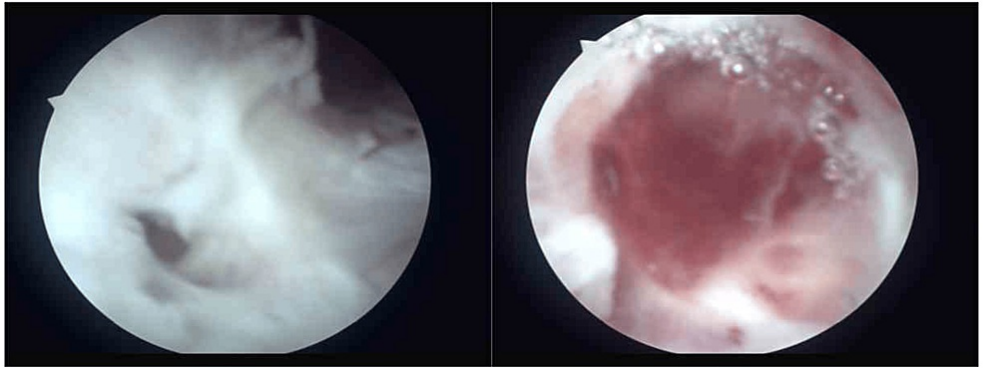
The cystoscopy examination revealed several mucosal tears within the bladder's right trigonal region and the absence of both the left ureteral orifice and the fistula. Additionally, there was a significant discharge of irrigation fluid through the vagina.

Cystoscopic examination revealed multiple mucosal disruptions in the bladder's right trigonal area and the absence of the left ureteral orifice and fistula. Notably, there was excessive exudation of irrigation fluid from the vagina (Figure 1). Further diagnostic imaging with MRI and ultrasound demonstrated a thickened bladder wall adversely affecting the left ureter, leading to hydronephrosis. PET-CT revealed no signs of cancer. The patient did not present with fever, cough, dyspnea, palpitations, nausea, vomiting, urinary frequency, urgency, dysuria, hematuria, or cloudy urine, and reported normal bowel movements, diet, sleep, and stable weight.

**Figure 2:**
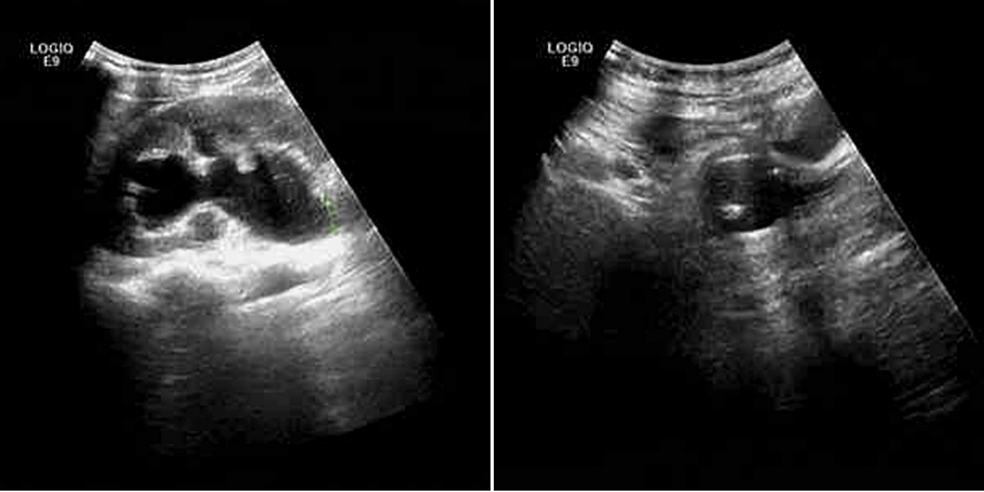
Ultrasound findings reveal a separation in the renal pelvis of the left kidney and dilation of the left ureter's upper segment.

The proposed management strategy included enhanced hydration and a three- to five-day course of anti-inflammatory treatment. Ultrasound imaging showed the left kidney measured 100×51 mm, with the right kidney slightly smaller at 99×47 mm. No abnormalities were observed in the right kidney sinus, and no ureteral dilation was noted. However, the left kidney exhibited a significant separation within the renal sinus, measuring 21 mm in width (Figure 2), and the upper segment of the left ureter was dilated, with an inner diameter of 9 mm. During examination, the bladder was not fully distended, but a catheter was in place.

**Figure 3:**
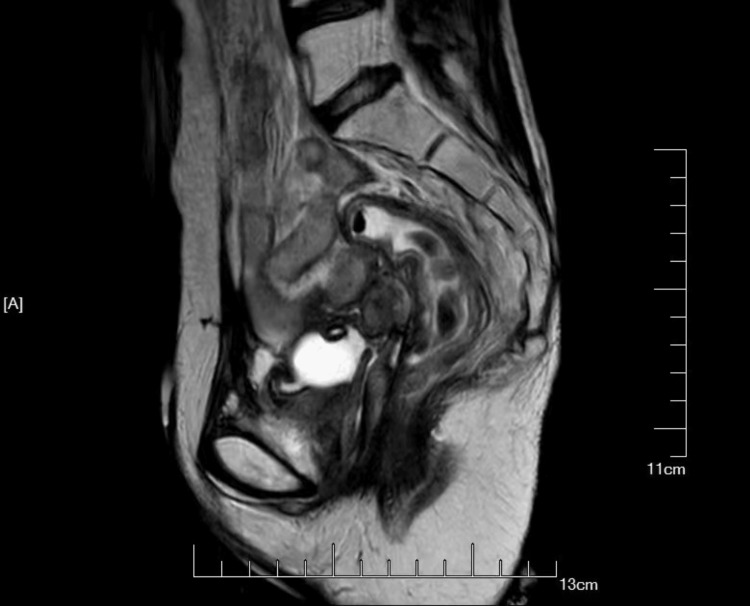
The MRI imaging revealed bladder wall thickening, which may extend to the left ureter. The enhancement of the vaginal stump appeared irregular, with indistinct separation from the bladder, suggesting the possibility of urine fluid leakage.

MRI findings indicated bladder wall thickening and left ureteral dilation, suggestive of inflammation, unclear visualization of the uterus and vaginal-urethral junction, a cystic signal abnormality in the left adnexal region, pelvic fluid collection, and mildly enlarged bilateral Inguinal lymph nodes, highlighting the intricacy of this case and the imperative for a meticulously crafted treatment approach (Figure 3).

The patient underwent laparoscopic surgery under combined anesthesia, targeting the removal of accumulated fluid, pus, and intestinal adhesions while elevating both ureters. Scar dissection was accomplished with minimal blood loss. The postoperative care regimen emphasized hydration and the administration of anti-inflammatory medications as listed in (Table 1). Subsequently, the patient developed a fever, initially managed with levofloxacin. Considering the persistence of symptoms, treatment options were expanded to include either piperacillin-sodium tazobactam or cefoperazone-sulbactam. Further details on the patient's response and ongoing management are outlined in laboratory test results presented in (Table 2).

**Table 1:** The medication table lists the several therapies that have been given to the patient, along with the individual medications and their dosages. It emphasizes the customized approach to therapy in accordance with accepted medical standards and gives a comprehensive summary of the therapeutic regimen utilized to manage the patient's symptoms and problems.

Medication Name	Dosage
0.9% Sodium Chloride Injection	250 mL
Levofloxacin Hydrochloride Injection	10 mL
5% Glucose Injection	500 mL
Potassium Chloride Injection	1.5 g
Flurbiprofen Axetil Injection	50 mg
Ibuprofen Injection	0.4 g
Piperacillin Sodium/Tazobactam Sodium Injection	4.5 g
Tropisetron Hydrochloride Injection	5 mg
Lipid Emulsion Amino Acid (17%) Glucose (11%) Injection	1440 mL
Potassium Citrate Granules	1.45 g

**Table 2:** The patient's test results, emphasizing anomalies such as low hemoglobin and red blood cells associated with anemia, high white blood cell counts suggesting infection or inflammation, and excessive bilirubin levels suggesting potential liver involvement.

Test	Reference range	Result
Total Bilirubin	3.4-20.5 µmol/L	29.3
Direct Bilirubin	0-3.4 µmol/L	11.0
Albumin	35-50 g/L	65.8
Total Protein	60-80 g/L	51.6
White Blood Cell Count	4.5-11.0 × 109/L	139.3
Red Blood Cell Count	4.3-5.9 x 1012/L	3.30
Hemoglobin	135-170 g/L	78
Platelet Count	150-400 × 109/L	986
Neutrophil Percentage	40-60%	80
Lymphocyte Percentage	20-40%	13
Monocyte Percentage	2-8%	3
Eosinophil Percentage	1-4%	0
Basophil Percentage	0.5-1%	0
Erythrocyte Sedimentation Rate	0-20 mm/h	80
Fibrinogen	2-4 g/L	4.69
Mean Platelet Volume (MPV)	7.5-11.5 FL	10.50
Platelet Distribution Width (PDW) Percentage	9-17%	20.2
Alkaline Phosphatase (ALP)	44-147 U/L	101.3
Gamma-Glutamyl Transferase (GGT)	10-71 U/L	134.3
Procalcitonin	0.5 ng/mL	0.550
Sodium	136-145 mmol/L	134.1
Calcium	2.2-2.6 mmol/L	2.09
Aspartate Transaminase AST	10-40 U/L	135.8
Hematocrit (HCT)	0.38-0.50 L/L	0.316
Mean Corpuscular Hemoglobin (MCH)	27-33 PG	83.4
Mean Corpuscular Hemoglobin Concentration (MCHC)	320-360 g/L	329
Globulin	20-35 g/L	17.9

**Figure 4:**
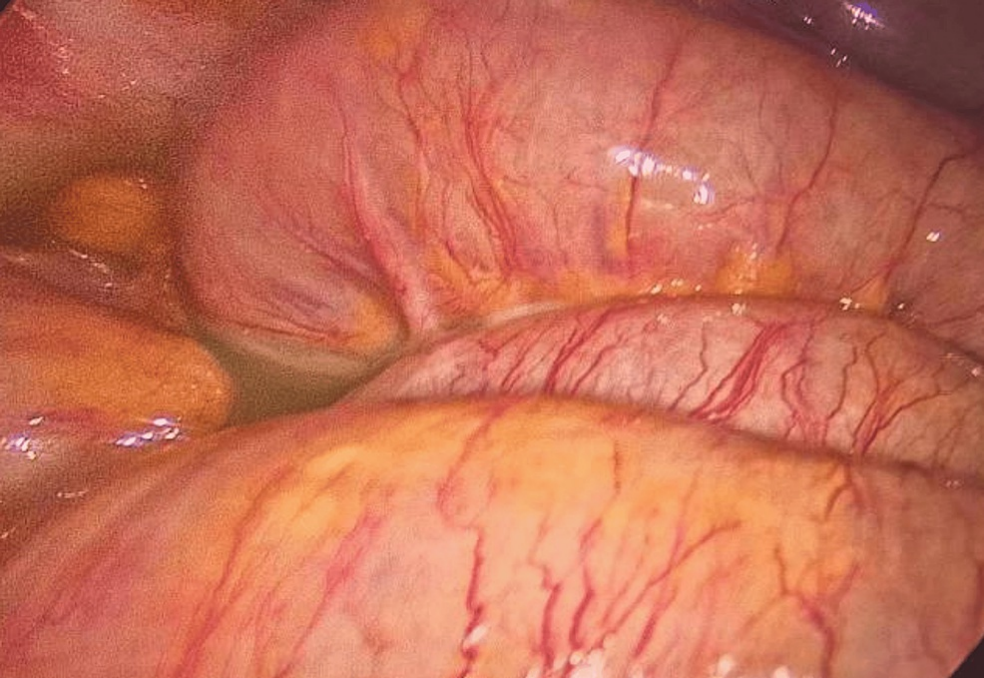
Purulent effusion in the pelvic cavity, attributed to urinary leakage, alongside notable intestinal edema and swelling, was observed.

Pelvic fluid accumulation, pus, urinary leakage, signs of infection, and intestinal swelling and edema in a patient previously treated with radiation for cervical cancer highlight the complex sequelae of pelvic radiation disease. This condition encompasses radiation-induced cystitis, VVF, and radiation enteritis, emphasizing the necessity of a multidisciplinary strategy. This approach is vital for navigating the delicate equilibrium between delivering effective cancer therapy and reducing the risk of long-term detrimental effects (Figure 4).

**Figure 5:**
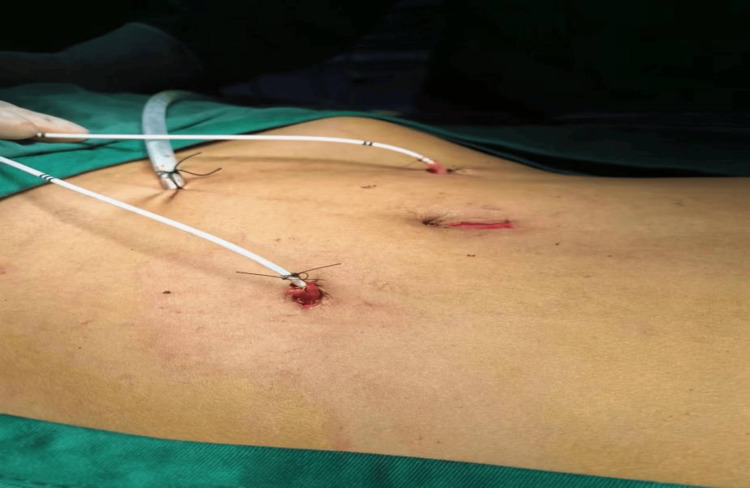
Both ureters are readily visible on postoperative imaging, with a good blood supply and ureteral ends. On the other hand, aberrant cutaneous connections are clearly seen, suggesting a substantial bilateral ureteral involvement.

Following surgery, postoperative imaging showed that both ureters were visible and appeared to be in good health, both the ureteral ends and the blood supply. Nevertheless, aberrant connections were also seen in the imaging that reached the skin, indicating a severe bilateral involvement of the ureters (Figure 5). This result emphasizes how serious the patient's condition is, how far the disease has spread, and how it affects the surrounding tissues.

## Discussion

Vesicovaginal fistulas are recognized as a complication subsequent to cervical cancer treatment, especially among patients who have undergone radiation therapy. Radiation exposure leads to increased tissue friability and diminished healing capabilities, thereby increasing the susceptibility to fistula development [4]. The complex interplay between prior radiation therapy may exacerbate the risk of developing fistulas, indicating a sophisticated pathophysiological process that necessitates further exploration.

Urogenital fistulas are categorized into four main types: vesico-uterine, vesicovaginal, urethro-vaginal, and uretero-vaginal. These fistulas can vary by location, number, size, and associated complications. Notably, 75% of VVF cases are attributed to complications following a transabdominal or transvaginal hysterectomy. Fistulas of small size, surrounded by quiet tissue and exhibiting minimal scarring, may close spontaneously [5,6].

Assessment of the size, quantity, and specific location of the fistula is critical prior to embarking on curative surgical interventions. Enhanced preoperative diagnostic measures contribute to more effective surgical planning. Typically, patients with a VVF post-operation can be readily identified due to urinary leakage via the vagina. Furthermore, a marked increase in leukocyte count may be present, indicating an immune response [7]. Fistulas typically manifest between the seventh and 12th day following obstetric or gynecologic surgery. Diagnosis can be determined by instilling the bladder with a diluted methylene blue solution. For patients exhibiting urinary incontinence, conducting a tampon test - where a tampon is inserted into the vagina after bladder instillation with the solution, followed by patient ambulation - may confirm the diagnosis. Cystoscopy serves as an invaluable diagnostic tool, offering precise identification of the fistula's anatomical origin. In cases of small fistulas, navigating a small ureteric catheter through the presumed fistula tract to verify its vaginal entry can be informative. A comprehensive physical examination is crucial, necessitating a meticulous inspection of the fistula site and its vicinity. Surgery should be deferred in the presence of acute inflammation, edema, necrosis, or concurrent bladder pathologies until these conditions are addressed. Preoperative assessments must consider any scarring at the fistula site, adhesion to neighboring organs, vaginal rigidity, or rectal involvement due to prior radiation, as these factors may necessitate modifications in the surgical strategy. Additional diagnostic measures should encompass retrograde and voiding cystourethrography to further elucidate the condition [7]. Therefore, ensuring an accurate diagnosis is crucial. The initial step involves a physical examination, specifically a pelvic exam to detect any lumps that may require further evaluation. Subsequently, a transvaginal ultrasound (TVUS) is employed as the first diagnostic tool. This imaging technique involves inserting an ultrasound probe into the vaginal canal to obtain images and examine the female reproductive organs [8]. TVUS has demonstrated a sensitivity of 97% and a specificity of 74% for identifying endometrial abnormalities [9]. If abnormalities are found, a biopsy is the next step. During a biopsy, a sample of cells is taken from the uterine lining and examined under a microscope. If endometrial cancer is detected, appropriate treatment will be administered. Conversely, if the biopsy results are normal or non-diagnostic, additional techniques can be employed to determine the cause of bleeding. For instance, color Doppler ultrasound can help differentiate between benign and malignant conditions, and saline contrast hysterosonography provides detailed imaging. Elevated creatinine levels in the discharge can substantiate the presence of urinary leakage. Nonetheless, intravenous pyelography and cystography might fail to reveal the genital anomalies [10]. More advanced, albeit invasive and potentially more expensive, techniques encompass the combined use of vaginoscopy and cystoscopy [11], subtraction magnetic resonance fistulography [12], and endocavitary ultrasound, conducted via the transrectal or, more appropriately, the transvaginal route, with the optional use of Doppler or contrast agents [7]. Transvaginal sonographic assessment offers precise visualization of the fistula's location, size, and trajectory. The literature describes it as well-tolerated, posing fewer risks, and providing more detailed information compared to traditional diagnostic methods [13]. Transvaginal sonography while offering detailed visualization of a fistula's specifics is dependent on the operator's expertise, potentially challenging for those less experienced compared to the traditional cystogram. In cases with suspected malignancy, histological examination through biopsies is imperative. Additionally, conducting an intravenous pyeloureterogram is advisable to exclude the presence of accompanying ureteral fistulas prior to surgical intervention.

Opting for laparoscopic surgery in the treatment of VVF, particularly given the patient's complex medical background and the procedure's minimally invasive characteristics, reflects the contemporary preference for reducing patient morbidity and facilitating quicker recovery [14]. The postoperative regimen, emphasizing hydration and anti-inflammatory medication, plays a pivotal role in addressing radiation cystitis and averting additional complications. The surgical resolution of VVF entails diverse techniques, whether through abdominal or vaginal routes. The choice of the most suitable method is informed by the individual characteristics of each patient. For patients with a considerable history of radiation, like in the discussed case, vaginal access might be compromised due to stricture development. The prevalence of radiation-induced vaginal stricture in individuals treated for cervical cancer exhibits a wide range, reported as anywhere between 1% and 88% [15].

The onset of fever post-surgery, along with laboratory signs of infection, inflammation, and anemia, poses considerable challenges in the postoperative care of patients with prior pelvic radiation exposure. Elevated total and direct bilirubin levels, together with alterations in white and red blood cell counts, highlight the systemic implications faced in the management of such complex cases. These findings emphasize the need for rigorous postoperative monitoring and the ability to adjust treatment plans according to evolving clinical and laboratory results.

This scenario underscores the critical need for customized, interdisciplinary care strategies that thoroughly consider the long-term impacts of cancer treatments. The development of a VVF after radiation therapy for cervical cancer underscores the delicate balance required to achieve cancer remission while preserving the patient's quality of life. Thus, this case contributes to the growing body of knowledge advocating for the integration of preventive actions and early diagnostic efforts in the treatment continuum for cancer survivors.

Given that the complications primarily result from the effects of radiation therapy, absent active cancer, and are seen rarely over a decade after treatment, standard procedures often focus on fistula repair. However, this case diverges from conventional treatment due to significant scar tissue in the bladder and radiation-induced fibrosis, leading to obstruction of the lower ureter, demanding a customized approach to manage these specific challenges.

This detailed narrative captures the complex clinical considerations and the precise approach needed in the care of long-term consequences of cancer treatment, promoting a strategic combination of alertness, flexibility, and comprehensive care in the postoperative phase.

## Conclusions

This case highlights the challenges of managing VVF and hydronephrosis as complications of radiation therapy for cervical cancer. The successful treatment involved a multidisciplinary approach, emphasizing the delicate balance between cancer treatment and preserving quality of life. The case underscores the importance of individualized care and the need for advanced management strategies to address the long-term consequences of cancer therapy, reaffirming the value of early intervention and personalized treatment plans in improving outcomes.
